# Supplementary material for: An essential role for maternal control of Nodal signaling
Source: eLife. 2013 Sep 10;2:e00683. doi: 10.7554/eLife.00683 (PMC3771576; doi:10.7554/eLife.00683)
Supplement: Supplementary file 1. — DOI: http://dx.doi.org/10.7554/eLife.00683.018 [file elife00683s001.docx]

**Supplementary file 1. Primer Sequence**s

| **Primer Name** | **Sequence** |
| --- | --- |
| For Templates to transcribe gel-shift probes: | |
| sqt.1T3-F | AATTAACCCTCACTAAAGGGAGATCATGAGACACCATGAAG |
| sqt.1-R | AAGGAGCATATCCAAAGTGC |
| sqt.2T3-F | AATTAACCCTCACTAAAGGGAGATTCTTCAAACCCCAAAG |
| sqt.2-R | AAGTGGGAATAATTGACAGC |
| sqt.3T3-F | AATTAACCCTCACTAAAGGGGACCCCAAAAATATGTAT |
| sqt.3-R | ATAGCATCAAGTTATCCAG |
| sqt.4T3-F | AATTAACCCTCACTAAAGGGAGAGAAATTATTATGGTTTC |
| sqt.4-R | CAGATAAGGCAAACACG |
| sqt.5T3-F | AATTAACCCTCACTAAAGGGAGATATTGAAAGCTTTGCGT |
| sqt.5-R | ATTATGAAAACATTTTATTAC |
| MmGAPDHT3-F | ATTAACCCTCACTAAAGGGAGATGAGAAACCCTGGACCACCCAC |
| MmGAPDH-R | CAGTGATGGGGGCTGAGTTG |
| gapdhT3-F | AATTAACCCTCACTAAAGGGAGAAAAGCCAGACCATTCCTTC |
| Gapdh-R | TTTTTAAACTGCATTACAGTAGCCTTT |
| cycT3-F | AATTAACCCTCACTAAAGGGAGAAGTGCGGATGCCTGTGA |
| cycT3-R | TGAGTGTGTGTTTGTGCGTC |
| wnt8a.1T3-F | AATTAACCCTCACTAAAGGGAGACCGGCCGCACAACCATTCAC |
| wnt8a.1-R | TATTTACATTAGAAATATAC |
| wnt8a.2T3-F | AATTAACCCTCACTAAAGGGAGATATTTTATGAGATTTTAAGA |
| wnt8a.2-R | GTGGGAACGAGAAAGCCCAT |
| wnt8a.3T3-F | AATTAACCCTCACTAAAGGGAGATGTCAATTGAATTCATTGAA |
| wnt8a.3-R | ACATTTTTTGAGAGCAACAA |
| wnt8a.4T3-F | AATTAACCCTCACTAAAGGGAGATTGTATTTTTTCATGCACAG |
| wnt8a.4-R | AAAATATTTGCCTTAAATA |
| vg1.1T3-F | AATTAACCCTCACTAAAGGGAGAAGTGGATGCAGATGAACATG |
| vg1.1-R | AAAAGAAGCCTAATTTTGC |
| vg1.2T3-F | AATTAACCCTCACTAAAGGGAGAACAATTTTTCTTTTTTTAGGTG |
| vg1.2-R | ATTATAAAAAGTTACTTTAACAGC |
| vg1.3T3-F | AATTAACCCTCACTAAAGGGAGAGATGCAGAGAATGTGC |
| vg1.3-R | GAAAAAAAAGGAATCCCATAGTAAAAG |
| For cloning Ybx1 and site-directed mutagenesis to generateYbx1 mutants: | |
| Ybx1-F | AAACACCATGGGCAGCGAGGCCGAGACACAACA |
| Ybx1-R | TGTTTAAGCTCGAGTAATCTGCTCCGCCCTGTTC |
| Ybx1-V83F-F | GAACAACCCCAGGAAATATCTCCGTAGCTTTGGGGACGGAGAG |
| Ybx1-V83F-R | CACGTCGAACTCCACAGTCTCTCCGTCCCCAAAGCTACGGAG |
| Ybx1-sg8-F | TACACCAACTCACAAAGAGGAGAGATGACAGGATCCAGATCTCAT |
| Ybx1-sg8-R | GTGATGGTGATGGTGATGAGATCTGGATCCTGTCATCTCTCC |
| Ybx1dssDBD-F | ATGGGCAGCGAGGCCGAGACACAAAGGAATGACACAAAG |
| Ybx1dssDBD-R | GCACAAAGACATCTTCCTTTGTGTCATTCCTTTGTGTCTCG |
| Ybx1dRNP-F | GTTTTGGGGACAGTGAAATGGTTCAATGTAAGGCAGACCGCCA |
| Ybx1dRNP-R | GAGATATTTCCTGGGGTTGTTCTTTTTAATGGCGGTCTGCCTTACATTG |
| Ybx1dCSD2-F | GGAATGACACAAAGGAAGATGTCTTTGTGCACGTTACCGGCC |
| Ybx1dCSD2-R | CTACCCTGCACAGGAACGCCACCCGGGCCGGTAACGTGCACAAAG |
| Ybx1dDIMER-F | GCCCGGGTGGCGTTCCTGTGCAGGGTAGTAAGTATAGCGACCCTG |
| Ybx1dDIMER-R | CTCTCTCTTCTCCCGGGGCTCTGCCTCAGGGTCGCTATACTTACTAC |
| Ybx1dCterm-F | CCACCTCGCGACTACCAGGAGAACTATCAGGGATCCAGATCTCATC |
| Ybx1dCterm-R | GTGATGGTGATGGTGATGAGATCTGGATCCCTGATAGTTCTC |
| Ybx1de8-F | GAACTACTACAGAGGCTTCCGACCAGGATCCAGATCTCATC |
| Ybx1de8-R | GTGATGGTGATGGTGATGAGATCTGGATCCTGGTCGGAAGC |
| Primers for sequencing ENU mutants | |
| forward outer primer | CAGGGATGGTAACTTTGCTC |
| reverse outer primer | AGGATTGAGTTTGACATCTGTG |
| forward inner primer with M13 forward tail | TGTAAAACGACGGCCAGT TCGGTGTAACCTGACTCTTG |
| reverse inner primer with M13 reverse tail | AGGAAACAGCTATGACCAT GCCTAATATTTCTAACTGTGTGGTG |
| RT-PCR and Q-PCR Primers: | |
| actinF | GGCTACAGCTTCACCACCA |
| actinR | TGCTGATCCACATCTGCTG |
| sqtF | GAACCACAGAACTGATGATA |
| sqtR | GCATGGTTTGTTGGAGTGAA |
| sqt-Ex2F1 | TGCCGAGCACTCCAAGTATG |
| sqt-Ex3R1 | CATCAAGTTATCCAGGTGCC |
| sqt-int1F | GTGGCCTGGGCATCAATACACAT |
| sqt-Ex2R | CAGGGGCTTCTCTCACACTC |
| sqt-Ex2F2 | GAAGGAACCACAGAACTGATGATA |
| sqt-Ex3R2 | GAGCATATCCAAAGTGCTAGAGTT |
| wnt8aF | AGTAATCCTCTTTGCAAATATGTAAAG |
| wnt8aR | AACCTCATCGTGAAACACTGC |
| gapdhF | GTTCATCCATCTTTGACGCTGGTGCTG |
| gapdhR | GAGGCCATGTGTGCCATCAGGTCA |
| gsc-F | TGGAAGGATAGGCTACAACAACTAC |
| gsc-R | GGTATTTCGTTTCTTGAAAAAGGTT |
| ntl-F | TATTGCAGTCACAGCATATCAGAAT |
| ntl-R | AAGCTGGAGTATCTCTCACAGTACG |
| bon-F | GAGAACTTACAAAGAACCTCAACATTTAC |
| bon-R | ACACTCAGGTGATCAGTTTTGATG |
| lft2-F | TTCATTACTGGTCTAAATCCCAAAA |
| lft2-R | CTCTGTCCATATCCATAGAAACCAC |
| bozF | GGCACTTGAGAAAGCTGGAC |
| bozR | GTAGTCGGTAACCGCGAAGA |
| voxF | GACCTCCGACATCATACGACAAG |
| voxR | CAGCGTCGTGTCCATCTTCG |
| spry4-F | CGGATAGACGTCCGCTTTTA |
| spry4-R | GGGGTGTCGATGTAGTCGTT |
| mxtx2-F | TCTGATCTGCAAGCAACACC |
| mxtx2-R | TGTCCCAAAATGCAGAATCA |
| hhex-F | ACCATCGAGCTGGAGAAGAA |
| hhex-R | GTCCTCCGCTTCCCTTTTAC |
| cldE-F | AGAGATTTCTACAATCCTCTGCTCA |
| cldE-R | GCTGGGAGTATTTCATGTTGTATTT |
| ybx1-F | GAGGGGGAGATGCAGCAGC |
| ybx1-R | TCTGCCTCATTGGTTTGTTG |
| Primer for genotyping *sqt^cz35^* allele | |
| sqtWT Fw | GAGCTTTATTTCAATAACTGCGTG |
| Cz35 Rev | ATATAAAATCAGTACAACCGCCCG |
| sqtWT Rev | GCCAGCTGCTCGCATTTTATTCC |
